# Cowden Syndrome Case Report: Use of an Ultrasonic Surgical Aspirator for Cosmetic Removal of Lip Hamartomas

**DOI:** 10.7759/cureus.29839

**Published:** 2022-10-02

**Authors:** William Montagne, Robert C Wang

**Affiliations:** 1 Otolaryngology - Head and Neck Surgery, University of Nevada Las Vegas School of Medicine, Las Vegas, USA

**Keywords:** cavitron ultrasonic surgical aspirator, ultrasonic surgical aspirator, cowden, hamartomas, lip hamartomas, cowden syndrome

## Abstract

Cowden syndrome (CS) is an autosomal dominant condition that is relatively rare. CS patients can have tumors derived from all three germlines. They can present with mucocutaneous hamartomas or other benign tumors, and have an increased risk of malignancies of the thyroid, breast, kidney, GI tract, and skin.

In our clinic, a 40-year-old CS patient presented for thyroidectomy after fine needle aspiration was suspicious for papillary thyroid carcinoma (PTC). Another major concern was the cosmetic appearance of her lips, which were diffusely covered with small hamartomas. We were able to remove these in a novel manner using a cavitron ultrasonic surgical aspirator (CUSA; Integra Lifesciences, Princeton, NJ, USA). Using the CUSA tangential to the lip surface allowed for removal of the hamartomas in a way that created a smooth and cosmetically appealing outcome for the patient. The use of an ultrasonic surgical aspirator is a novel way to cosmetically treat hamartomas of the lip for CS patients.

## Introduction

Cowden syndrome (CS) is a phosphatase and tensin homolog (PTEN) hamartoma syndrome. It affects approximately 1 in 200,000 individual births, but is thought to be underestimated due to its variable penetrance [1,2]. CS is inherited in an autosomal dominant pattern. CS is characterized by multiple hamartomatous neoplasms of ectodermal, mesodermal and endodermal origin [3-5]. The clinical features of CS commonly include benign breast disease, benign thyroid nodules and mucocutaneous lesions. CS also carries increased risk of malignancies in all three germ layers. CS patients carry a lifetime risk of breast cancer ~85%, thyroid cancer ~38%, endometrial cancer ~28%, colorectal cancer ~9%, and melanoma ~6% [4,5]. Due to the PTEN germline mutation, CS patients can present in a variety of ways to a variety of specialists. The vast majority of CS patients have mucocutaneous hamartomas by the third decade of life [5,6]. Although these are benign lesions they can cause symptoms including pain, discomfort and emotional hardship due to cosmesis. These lip hamartomas have been removed with various techniques including cold steel and cautery [7-9]. These techniques can allow for the removal of a single or multiple hamartomas, but can present difficulty in achieving good cosmetic results.

In this case report, we present a novel technique for removing lip hamartomas with a satisfactory outcome for the patient.

## Case presentation

A 40-year-old female with a history of CS presented to our clinic for thyroidectomy after fine needle aspiration results suggested papillary thyroid carcinoma (PTC). During her clinic visit and examination she was found to have diffuse mucocutaneous hamartomas and an atypical appearing nevus on her neck. She reported emotional distress from the appearance of her lip hamartomas and discomfort. She wanted those removed in addition to her thyroid. A surgical plan was made for a total thyroidectomy, removal of skin nevus and removal of benign lip lesions.

**Figure 1:**
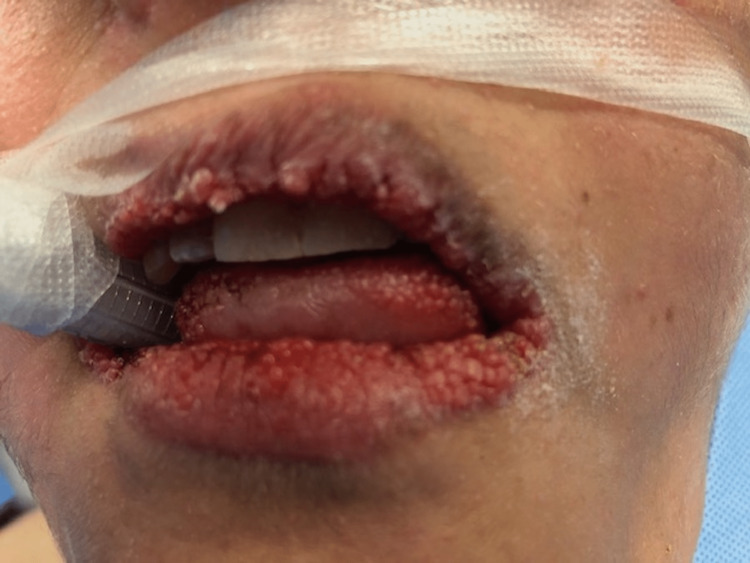
Pre-operative image of lips showing diffuse small hamartomas.

**Figure 2:**
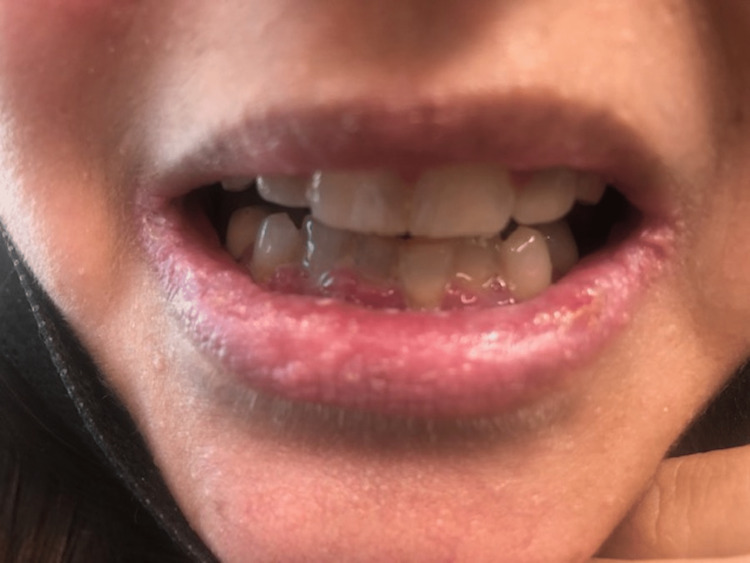
Ten-day post-operative image of lips showing hamartomas removed.

**Figure 3:**
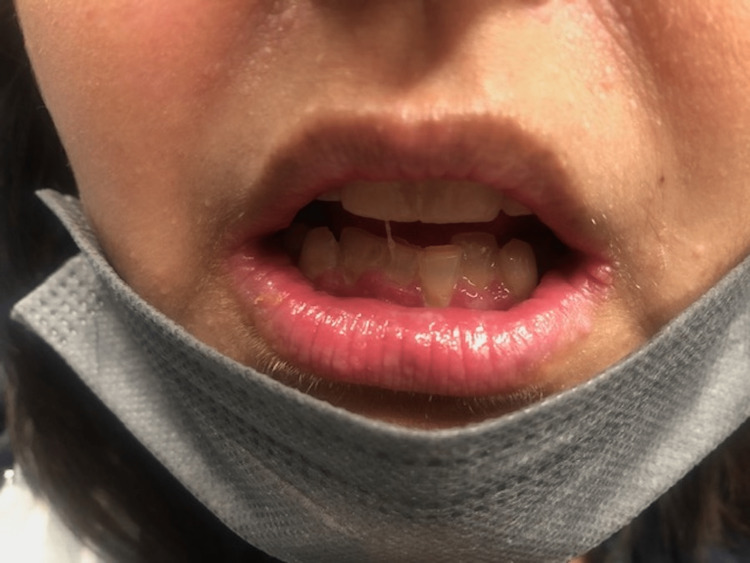
Three-month post-operative image of lips showing interval healing.

**Figure 4:**
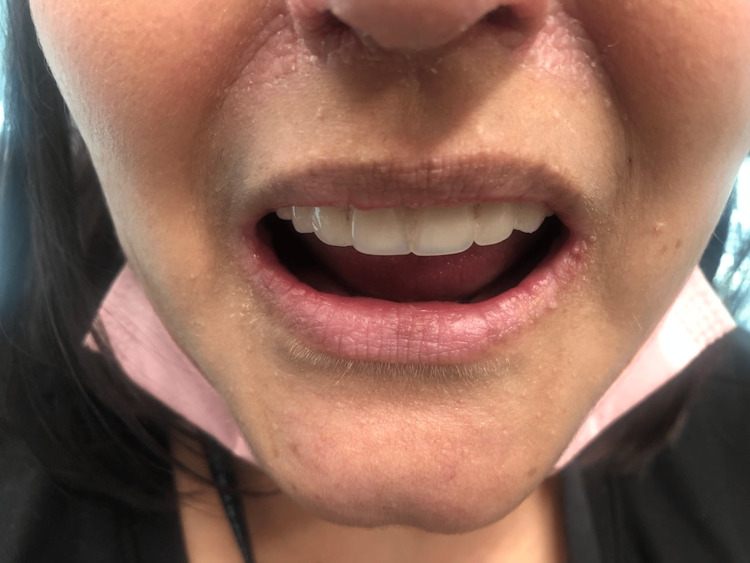
Nine-month post-operative image of lips with continued healing and good cosmetic outcome.

She was taken to the operating room with an uneventful removal of her thyroid and skin lesion. The final pathology showed that the right thyroid lobe had a 1.3 cm PTC follicular variant and 0.5 cm PTC classic variant BRAF+. The left thyroid lobe had a 0.6 cm PTC follicular variant and a 0.3 cm PTC follicular variant. The cutaneous lesion on the neck was an atypical nevus. Focus was then placed on the lip hamartomas, pre-op picture in Figure 1. An initial attempt was made using electrocautery to remove an inner lip hamartoma, but this left a large depression and charring and would potentially heal with an uneven contour and submucosal scarring. We then switched to a cavitron ultrasonic surgical aspirator (CUSA; Integra Lifesciences, Princeton, NJ, USA). Setting the device with an amplitude of 75%, tissue select of 3 (high), aspiration of 100% and irrigation of 8 ml/min was found to be effective at removing the hamartomas. Our technique used the off-hand and a 4x4 to hold tension on the lips. Then using the CUSA in a tangential fashion to the lip surface, the hamartomas were removed without creating divots in the lips. This was done to remove all the hamartomas from the lips. We then used epinephrine-soaked gauze to obtain hemostasis. Pathology from the lip tissue returned as benign hamartomatous tissue. Her hospital course was uncomplicated and she was discharged. Post-operative care for her lips included Vaseline or Aquaphor as needed to keep them moist and prevent crusting. She returned on postoperative day 10 for follow-up. At that time she was satisfied with the cosmetic outcome and feeling of her lips (Figure 2). At three months, her lips were smooth in contour and soft to palpation. She continued to be pleased with the appearance and consistency of her lips (Figure 3), allowing her to kiss her children and spouse without feeling self-conscious anymore. At nine months, she continued to report satisfaction with her lips (Figure 4). Although asymptomatic, there appeared to be a small hamartoma at the left oral commissure. We plan to continue to monitor and follow her for both her thyroid cancer and any recurrence of her lip hamartomas.

## Discussion

Cowden syndrome is characterized by neoplasms of ectodermal, endodermal and mesodermal origin. These patients usually develop lip hamartomas by the third decade of life [2]. Although these hamartomas are benign lesions, they can cause discomfort and emotional distress due to cosmetic appearance. These mucocutaneous lesions have been removed with different surgical techniques including cold surgery, cautery, laser and cryosurgery [7-9]. Here we presented a novel technique using an ultrasonic surgical aspirator to remove lip hamartomas with satisfactory cosmetic outcomes.

CS was first described in 1963 by Lloyd and Dennis about a patient named Rachel Cowden [10]. In 1972 the presence of CS was confirmed by Weary et al. and in 1996 the International Cowden Consortium identified mutations in the PTEN gene on chromosome 10 as the cause of CS [2,11]. CS is now well recognized as a highly variable autosomal dominant hereditary cancer susceptibility syndrome. It is characterized by multiple hamartomas and increased risk of developing malignancies in all three germ layers. CS typically presents in the second to third decade of life but has been diagnosed anywhere from four to 75 years of age. It is slightly more common in females than males [4,12].

Updated diagnostic criteria for CS were developed by Pilarski et al. in 2013 and recognized by the National Comprehensive Cancer Network with guidelines released in 2021 [13]. Diagnostic criteria are broken down into major and minor criteria. Major criteria include: breast cancer, endometrial cancer, thyroid cancer (follicular), GI hamartomas, Lhermitte-Duclos disease, macrocephaly, macular pigmentation of the glans penis and multiple mucocutaneous lesions. Minor criteria include autism spectrum disorder, colon cancer, esophageal glycogenic acanthoses, lipomas, intellectual disability, renal cell carcinoma, testicular lipomatosis, thyroid cancer, thyroid lesions and vascular anomalies. Diagnosis is made with three or more major criteria or two major and three minor criteria being met.

CS affects all germ layers and can present with benign or malignant lesions in a variety of anatomical locations. Breast cancer is the most common malignancy in CS with lifetime risk of up to 85% compared to about 12-13% in the general population [5,14,15]. Benign breast lesions affect about 50% of CS patients [9]. CS also affects the central nervous system and can result in Lhermitte-Duclos disease (LDD), macrocephaly and developmental issues. LDD is a benign lesion of the cerebellum. LDD can affect up to 40% of CS patients and is pathognomonic of CS in adults [5,15]. CS patients have GI involvement in 70-85% of cases. These are usually benign lesions with GI polyps anywhere from the esophagus to the rectum. CS carries a lifetime risk of colorectal cancer of about 9% [5,6]. CS can affect the genitourinary system in both males and females. It carries a lifetime risk of endometrial cancer of 28% compared to about 2-4% in the general population [5,15]. Lifetime risk of renal cell carcinoma in CS is around 33% [5,9]. CS patients carry an increased lifetime risk of melanoma at 6% compared to 2% in the general population [5,16]. Vascular malformations occur in 18-34% of CS patients compared to 5-10% of the general population [17]. Thyroid cancer is the second most common cancer in CS patients. Estimated lifetime risk of thyroid cancer is 38% in CS patients compared to <1% in the general population. Benign thyroid lesions are reported in 50-70% of CS patients [5,16,18]. Mucocutaneous lesions occur in a majority of CS patients (almost 100%) and these are usually benign [5,6]. Due to the diffuse anatomical involvement of CS, these patients can present in a variety of clinics. Therefore physicians should be familiar with CS and consider this diagnosis for patients with multiple benign or malignant lesions. CS patients require multidisciplinary care for both management and surveillance.

The technique we describe above may be limited as it has only been used on one patient, but it seems effective in achieving smooth contouring of the lips without deep scarring. To our knowledge there have not been reports of recurrence after removal of lip hamartomas in the literature, although most reports only have short follow up periods. Our study is also limited in determining the cost-effectiveness of this technique compared to other possible techniques. To determine effectiveness and long-term outcomes of this technique there needs to be further analysis including more patients and longer follow up. 

## Conclusions

A majority of CS patients develop mucocutaneous hamartomas by the third decade of life. Although these are benign lesions they can cause discomfort and be emotionally distressing to patients. These have been removed with a variety of surgical techniques typically due to discomfort. Here we present a novel technique to remove lip hamartomas due to cosmetic distress and discomfort. In our patient, the use of an ultrasonic surgical aspirator allowed for removal of diffuse lip hamartomas with satisfactory outcomes. Further studies need to be done to determine if this technique can be reproduced and has satisfactory long-term outcomes.
